# Intrahepatic Cholestasis of Pregnancy: Spontaneous *vs in vitro* Fertilization

**DOI:** 10.5005/jp-journals-10018-1232

**Published:** 2017-09-29

**Authors:** Filiz F Bolukbas, Cengiz Bolukbas, Hatice Y Balaban, Cem Aygun, Seyda Ignak, Emine Ergul, Mehtap Yazicioglu, Suat S Ersahin

**Affiliations:** 1 Department of Gastroenterology, Bahcesehir University School of Medicine, Medicalpark Goztepe Hospital, Istanbul, Turkey; 2 Department of Gastroenterology, Istanbul Medipol University School of Medicine, Istanbul, Turkey; 3 Department of Medical Biology, Bahcesehir University School of Medicine, Istanbul, Turkey; 4 Department of Gynecology and Obstetrics, Istanbul Bahcelievler Medicalpark, Istanbul Turkey; 5 Department of Gynecology and Obstetrics, Medicalpark Goztepe Hospital, Istanbul, Turkey

**Keywords:** Bile acid, *In vitro* fertilization, Intrahepatic cholestasis of pregnancy, Pruritus, Stillbirth.

## Abstract

**Aim:**

Intrahepatic cholestasis of pregnancy (ICP) is the most common liver disease in pregnancy. Although it was shown that multiple pregnancy and hormone therapies increase the risk of ICP, there is limited information that compared spontaneous fertilization and *in vitro* fertilization (IVF) from the aspect of developing ICP. In our study, we investigated the potential relationship between ICP and IVF/ spontaneous pregnancy.

**Materials and methods:**

We reviewed the records (between June 2007 and December 2014) of pregnancies with ICP who were referred to gastroenterology clinics in three different hospitals. Fifty-nine pregnancies (43 spontaneous fertilization, 16 IVF) with ICP were analyzed from the aspect of age, fertilization type, multiple/singleton pregnancy, delivery week, and biochemical results.

**Results:**

We found that serum bile acid levels were higher in the IVF group than the spontaneous fertilization group (32.8 ± 20 *vs* 19.6 ± 19 μmol/L; p < 0.05). There was a significant inverse correlation between serum bile acid levels and gestational age (r = -0.42, p < 0.01) in the whole group. There was no difference between IVF and spontaneous fertilization groups in term of age, onset time of symptoms, serum alanine aminotransferase (ALT), alkaline phosphatase (ALP), total and direct bilirubin levels, prothrombin time (PT), international normalized ratio (INR), and platelet count.

**Conclusion:**

Our results suggest that the serum bile acid levels are higher in IVF than in spontaneous pregnancies with ICP, but its clinical implications are not clear. Further prospective studies with large number of ICP cases are needed to clarify the effect of IVF on ICP.

**How to cite this article:** Bolukbas FF, Bolukbas C, Balaban HY, Aygun C, Ignak S, Ergul E, Yazicioglu M, Ersahin SS. Intrahepatic Cholestasis of Pregnancy: Spontaneous *vs in vitro* Fertilization. Euroasian J Hepato-Gastroenterol 2017;7(2):126-129.

## INTRODUCTION

Intrahepatic cholestasis of pregnancy is a pregnancy-specific liver disease, characterized by maternal pruritus and raised serum bile acids. It is the most common liver disease of pregnancy. Some studies showed that there is higher risk in women who have conceived by IVF (2.7% IVF treatment *vs* 2% spontaneous fertilization) and in those older than 35 years.^1,2^

The ICP is also associated with increased incidence of some fetal complications, such as fetal distress, premature delivery, and stillbirth.^3,4^ On the contrary, there is evidence that ICP increases the risk of adverse perinatal outcome, and there is no evidence that ICP increases the risk of stillbirth.^5,6^ There is a positive correlation between the rates of fetal complications and the level of total bile acids in maternal serum.^7-10^ A larger study of Swedish ICP cases showed that preterm labor was significantly higher in ICP pregnancies whose fasting serum bile acid level exceeds 40 μmol/L. It is thought that fetal complications did not arise until bile acid level increases above 40 μmol/L.^7^ The liver transaminases are also elevated in the majority of ICP patients. This may occur before or after the rise in serum bile acids. Serum ALT is more sensitive than aspartate transaminase (AST) in the diagnosis of ICP and may be raised 2- to 30-fold.^4^

Although it was shown that multiple pregnancy and hormone therapies increase the risk of ICP, there has not been a comprehensive research that compares with spontaneous fertilization and IVF from the aspect of developing ICP. In our study, we investigated the potential relationship between ICP and IVF *vs* spontaneous pregnancy by evaluating the health condition of pregnant and newborn patient records respectively.

## MATERIALS AND METHODS

This study was conducted in accordance with revised Helsinki Declaration and approved by the medical ethics committee. We retrospectively reviewed the records of pregnant women who were referred for various reasons to gastroenterology clinics from three different general hospitals (each of them has more than 200 bed capacity and more than 1,000 outpatients/day). Pregnant women with ICP were included in this study; ICP was defined as presence of pruritus without a rash, together with raised level of serum bile acids (cut-off level 10 μmol/L) and/ or raised level of serum ALT (>40 U/L). The exclusion criteria were extrahepatic biliary tract obstructions, viral or autoimmune liver diseases, HELLP syndrome (hemolysis, elevated liver enzymes, low platelet count), and fatty liver of pregnancy.

The records were reviewed from the aspect of pregnancy age, fertilization type (spontaneous or IVF), multiple/singleton pregnancy, gestational age, starting week of pruritus, drug usage during pregnancy, ICP history at previous pregnancy or within the family, type of delivery (spontaneous or induced), and maternal (mortality and postpartum hemorrhage) and fetal complications (fetal distress, congenital anomalies, respiratory distress). Spontaneous preterm delivery was defined by delivery occuring at gestational age between 31 and 36 weeks without any medical intervention. The delivery induction policy in ICP pregnancy was the first sign of fetal distress or reaching 37 weeks of pregnancy.

Complete blood count levels were determined by using Sysmex XN-1000 (Kobe, Japan) analyzer. Routine biochemical tests, such as fasting blood glucose, serum creatinine, uric acid, AST, ALT, ALP, gamma-glutamyl transferase, total protein, albumin, total bilirubin, direct bilirubin levels, and viral serology (Toxoplasma immunoglobulin [Ig]G, Toxoplasma IgM, Rubella IgG, Rubella IgM, Cytomegalovirus IgG, Cytomegalovirus IgM, Herpes simplex virus Type I IgG, Herpes simplex virus Type I IgM, Epstein-Barr virus [EBV] viral capsid antigen [VCA] IgM, EBV VCA IgG, Hepatitis B surface antigen, antihepatitis C virus, antihuman immunodeficiency virus 1,2) were determined by Abbott ci-8200 (Abbott Laboratories, IL) autoanalyzer. Prothrombin time and INR levels were determined using STA Compact (Diagnostica Stago, France). Serum bile acid levels were determined by radioimmunoassay. Autoimmune markers (antinuclear antibody, antimitochondrial M2) were determined using enzyme immunoassay method. All patients had the treatment with ursodeoxycholic acid (UDCA, Dr. Falk Pharma GmbH, Freiburg, Germany) 10 to 15 mg/kg/ day. The periodical follow-ups were done for women by symptom questioning, biochemical tests (serum bile acids, ALT, PT-INR) and for fetuses by ultrasonography (heart beats and development rate).

### Statistical Analysis

The data analysis was conducted with the statistical package, PSPP (psppire data editor XQuartz 2.3.6.xorg-server 1.4.2-apple56, Free Software Foundation, Boston, USA) for MAC Pro X, below the 0.05 level of significance. We analyzed the data at 95% confidence interval for mean with standard deviation. Nonparametric Pearson chi-square test and independent samples t-test were used for comparison between means and percentages. Correlation was done with 2-tailed "Pearson correlation test."

## RESULTS

A total of 59 pregnant women with ICP were recruited for this study (43 spontaneous fertilization and 16 IVF) between June 2007 and December 2014.

Progesterone was used in case of threatened miscarriage in 23% of spontaneous pregnancies. Multiple pregnancies were more common in the IVF than spontaneous fertilization (81 *vs* 4.6%, p < 0.01). Mean maternal age, onset time of symptoms, serum ALT, ALP, total and direct bilirubin levels, PT-INR, and platelet count were similar in IVF and spontaneous fertilization groups (Table 1). However, the serum bile acid levels were higher in IVF group than in spontaneous fertilization group (32.8 ± 20 *vs* 19.6 ± 19 μmol/L, p < 0.05).

There was a significant inverse correlation between maternal age (mean 30.2 ± 4 years) and onset time of symptoms in overall pregnancies (r = -0.31, p < 0.05). But fertilization type did not affect the onset time of symptoms. The location of pruritus was as follows: palm and sole (52%), extremities (20%), extremities and body (10%), only lower extremities and body (9%). There was no clinical jaundice, although serum total bilirubin levels were elevated (>1.2 mg/dL) in 8.5% of whole group. Splenomegaly was not found in any pregnant woman on physical examination. The maternal age at the time of delivery was 30.24 ± 4.2 years, and was similar in spontaneous fertilization and IVF groups. Multiple pregnancies had earlier delivery (singleton 37 ± 1.8 *vs* multiple 34 ± 2.2 weeks, p < 0.01). The spontaneous preterm delivery was more frequent in IVF than spontaneous fertilization group (43.75 *vs* 25.58%, p < 0.05), as well as multiple pregnancies than singleton (53.33 *vs* 22.73%, p < 0.05). Additionally, there was an inverse correlation between serum bile acid levels and time of delivery in overall pregnancies (r = -0.42, p < 0.01). Eight pregnant women waited for spontaneous labor until 40 weeks of gestation and all of them had singleton fetus with spontaneous fertilization. There was no maternal mortality or postpartum hemorrhage. After the delivery, only 70% (45/59) women could be followed up. The serum bile acid and ALT levels decreased to the normal level, and pruritus dramatically regressed within the first week after birth.

The fetal distress was detected in two of spontaneous fertilization group and three of IVF group. There was only one stillbirth at 37 weeks in spontaneous fertilization group. In this case, the serum bile acid level was 21.5 μmol/L, and the family did not accept the necropsy. There were no congenital anomalies in both groups.

**Table 1:** **Table 1:** Comparison of spontaneous fertilization and IVF groups

		*Spontaneous group (n = 43)*		*IVF group (n = 16)*		*p-value*	
Age of pregnant women (years)		29.9 ± 4.7		30.9 ± 2.5		0.44	
Onset time of symptoms (weeks)		28.1 ± 9.4		29.6 ± 6.2		0.57	
Multiple/singleton (n)		2/41		13/3		0.001	
Serum ALT (IU/L)		173 ± 193		240 ± 375		0.38	
Serum ALP (IU/L)		225 ± 114		217 ± 92		0.82	
Total bilirubin (mg/dL)		0.8 ± 0.5		0.8 ± 0.3		0.74	
Platelet count (10^3^/μL)		237 ± 57		229 ± 104		0.73	
PT (seconds)		10.86 ± 1		11.66 ± 0.8		0.06	
PT-INR		1 ± 0.13		0.95 ± 0.9		0.24	
Serum bile acid (μmol/L)		19.6 ± 19.0		32.8 ± 19.0		0.05	
ICP history during previous pregnancy		23.2%		6.25%		0.05	
Family history for ICP		20.9%		25.0%		0.44	
Using hormonal drug during pregnancy		23%		100%		0.001	
Gestational age at delivery (weeks)		37.0 ± 2.2		35.0 ± 2.3		0.10	

## DISCUSSION

This study investigated the relationship between ICP and fertilization type by retrospective analysis of 59 pregnancies with ICP. The serum bile acid levels were higher in IVF than spontaneous pregnancies and were inversely correlated with time of delivery. The increased serum bile acid levels in the IVF pregnancies could be explained by higher rates of multiple pregnancy and hormone treatment. The maternal and fetal outcomes of pregnancies were similar in both groups.

The incidence of ICP can be as high as 4%, although it varies widely with geographical location and ethnicity.

The ICP is most common in South America and Northern Europe.^3^ The incidence of ICP has been reported to be up to 20 to 22% in multiple pregnancies.^4^ The etiology of ICP is complex and not fully understood, but it is thought that abnormal biliary transport may be the result of a number of factors including hormonal, environmental, and genetics.^11^

A pregnant woman with typical pruritus is considered to have ICP in the absence of evidence for an alternative diagnosis, such as viral or autoimmune hepatitis, extrahepatic biliary tract obstructions, and fatty liver of pregnancy.^4,12^ The most sensitive and specific marker for diagnosis of ICP is the serum bile acid level. The ICP is associated with the rise of serum bile acid levels to ≥10 μmol/L.^7-10^ Although there is no concensus on cut-off value, most studies for ICP define the upper limit of normal serum bile acid level as 10 μmol/L.^7,11^ We defined ICP as having pruritus without a rash, together with raised level of serum bile acids (10 μmol/L) and/or raised level of serum ALT (> 40 U/L) in this study.

The ICP cases have been reported as early as 8 weeks until as late as 35 weeks.^4^ In our study, onset of symptoms was at 28 to 29 weeks, and there was no difference according to fertilization type. Bilirubin is normal in the majority of ICP cases and is of limited value in diagnosis.^13^ Hyperbilirubinemia in ICP is rare, affecting approximately 10 to 15% and it also tends to be mild.^14^ Although there was no clinical jaundice in the whole group, serum total bilirubin levels were elevated (>1.2 mg/dL) in 8.5% of pregnant women. The exact cause of hyperbilirubinemia in ICP is unknown but is speculated that hormones might play a role.

Stillbirths in ICP cluster around 37 to 39 weeks.^7,15-18^ Perinatal fetal mortality related to ICP has been reduced 3.5% or less in more recent studies employing policies of active management.^2,4,16,19^ The IVF pregnancies also carry increased risk of complications related with maternal characteristics, such as age, infertility, and multiple pregnancies.^1^ In our study, spontaneous preterm delivery is very high especially in multiple pregnancies, and there was one stillbirth at 37 weeks in a woman with spontaneous pregnancy and serum bile acid level of 21.5 μmol/L. We thought our findings on complication of ICP is compatible with literature.

There is a support for use of UDCA in women with ICP because of improvement in maternal pruritus, although definitive evidence for fetal benefit is still lacking.^20-22^ We used UDCA in all pregnancies with ICP.

## CONCLUSION

We found that serum bile acid levels were higher in the IVF group than the spontaneous fertilization group. But fertilization type did not affect the onset time of symptoms. There was a significant inverse correlation between serum bile acid levels and gestational age in both spontaneous and IVF groups. We think that our study has some limitations. The small sample size might lead to type II statistical error and might be the reason for lack of correlation between type of fertilization and ICP. Additionally, the confounding factors, such as multiple pregnancies might affect the results, such as higher serum bile acid levels and earlier delivery in IVF group. The retrospective, cross-sectional study design also did not allow a cause-and-effect analysis. Further prospective studies with large number of ICP cases are needed to clarify the effect of IVF on ICP. In conclusion, the serum bile acid levels are higher in IVF than spontaneous pregnancies with ICP but its clinical implications are open to research.
